# Minimizing Contradictions on Circular Order of Phylogenic Trees

**Published:** 2007-10-11

**Authors:** Marc Thuillard

**Affiliations:** Belimo Automation AG, CH-8340 Hinwil, Switzerland.

**Keywords:** phylogenetics, circular order, distance-based estimation, lateral gene transfer

## Abstract

Distance-based approaches to phylogeny use estimations of the evolutionary distance between sequences to reconstruct an evolution tree. If the evolution can be represented by an X-tree, the different sequences can be ordered so that the distance matrix $Y_{i,\;j}^{n}$, representing the distance from a leaf *n* to the path (*i, j*), is perfectly ordered meaning that $Y_{i,\;j}^{n}\;\geq \;Y_{i,\;k}^{n}$ and $Y_{k,\;j}^{n}\;\geq \;Y_{k,\;i}^{n}$ for *i* ≤ *j* ≤ *k*. After ordering of the sequences, the distance matrix $Y_{i,\;j}^{n}$ permits to visualize phylogenetic relationships between taxa and to localize deviations from perfect order. The effect of perturbations resulting from lateral gene transfer or crossover can be modeled probabilistically. The order is shown to be quite robust against many perturbations. We have developed algorithms to minimize the level of contradiction in the order of the sequences. These algorithms are tested on the SSU rRNA data for Archaea. The degree of contradiction after optimization is for most taxa quite low. Regions in the taxa space with deviations from perfect order were identified.

## 1. Introduction

The most popular method to reconstruct an evolution tree from distances between sequences is the Neighbor-Joining algorithm (NJ) first developed 30 years ago by Saitou and Nei (1987). The NJ method has become theoretically better understood in very recent years. One can now explain why the method does work practically so well and why it is, in several instances, optimal (Bryant, 2005; Gascuel and Steel, 2006; Mihaescu et al. 2006).

Some studies (Yushmanov, 1994; Makarenkov and Leclerc, 1997, 2000; Korostensky and Gonnet, 2000; Semple and Steel, 2004) focused on an interesting property of so-called X-trees, namely that the order at which leaves are encountered on a tree must correspond to one of the possible compatible orders, called circular orders. Semple and Steel (2003) have characterized the number of circular orders for which two leaves are consecutive and related that number to an expression of the tree length given by Pauplin (2000). The so defined tree length turned out to be a central element in understanding the NJ algorithm: The NJ is a greedy algorithm that selects at each step as neighbors the pair of current taxa which most decreases an estimate of the whole tree length (Gascuel and Steel, 2006). The better understanding of the reconstruction of a tree, fitting best a dissimilarity matrix, has lead to new algorithms such as BioNJ or FASTME (Gascuel, 1997; Desper and Gascuel, 2002).

The paper addresses the problem of determining a circular order when the dissimilarity matrix corresponds only approximatively to an X-tree. We define optimality criteria and develop efficient algorithms to minimize the deviation of the best order to a circular order. Beyond furnishing a candidate best solution to the ordering problem, the algorithms provide a new way to visualize phylogenetic data in a simple 2-dimensional representation.

The outline of the paper is the following. In the second section, two methods for ordering X-trees are explained. We show that when the elements are circularly ordered, all elements of a transformed distance matrix obey simple inequalities. In the third section, we will explain how deviations from a perfect X-tree, due to lateral transfer or crossover, affect the order of the leaves. The first three sections build up the theory necessary to the understanding of the *minimum contradiction* approach at the heart of the ordering algorithms described in Section 4. In the last section, the algorithms are tested on the SSU rRNA data for Archaea.

## 2. Circular Ordering = Ordering of a Transformed Distance Matrix

### 2a. Preliminaries and definitions

Let us start by recalling a number of definitions that are necessary to introduce the notion of circular order. A graph *G* is defined by a set of vertices *V* (*G*) and a set of edges *E* (*G*). Let us write *e* (*x*, *y*) the edge between the two vertices *x* and *y*. In a graph *G*, a path *P* between two vertices *x* and *y* is a sequence of non-repeating edges *e* (*x*_1_, *z*_1_), *e* (*z*_1_, *z*_2_), …, *e* (z*_i_* *, y*) connecting *x* to *y*. The degree of a vortex *x* is the number of edges *e* ∈ *E* (*G*) to which *x* belongs. A leaf *x* of a graph is a vortex of degree one and an internal vortex is a vortex of degree larger than one.

A valued X-tree *T* is a graph with *X* as its set of leaves and a unique path between any two distinct vertices *x* and *y*, with internal vertices of at most degree 3. The distance *d* between leaves satisfies the classical triangular inequality.

(1) d(x, z) ≤ d(x, y) + d(y, z) for all x, y, z ∈ X

with *d* (*x*, *y*) representing the sum of the weights on the edges of *T* in the path connecting *x* and *y*.

A central problem in phylogeny is to determine if there is an X-tree *T* and a real-valued weighting of the edges of *T* that fits a dissimilarity matrix δ. Typically, a dissimilarity matrix δ corresponds to an estimation of the pairwise distance *d* (*x*, *y*) between all elements in *X*.

A necessary and satisfactory condition for the existence of a unique tree is that the dissimilarity matrix δ satisfies the so-called four-point condition (Bunemann, 1971). For any four elements in *X*, the 4-point condition requires that

(2) $$\begin{array}{c} \delta (x_{1},\;x_{2})\;+\;\delta (x_{3},\;x_{4})\;\leq \;\text{max}(\delta (x_{1},\;x_{3})\\ +\;\delta (x_{2},\;x_{4}),\;\delta (x_{1},\;x_{4})\;+\;\delta (x_{2},\;x_{3})) \end{array}$$

In this article, we will address the following problem. Considering an arbitrary dissimilarity matrix δ on *X*, determine if there is an order of the elements in *X* consistent with an X-tree *T*. In order to precise the problem, we will have to introduce the notion of circular order.

Consider a graphic planar representation of a tree *T*. A circular order (Barthélemy and Guénoche, 1991) corresponds to an indexing of the *n* leaves according to a circular (clockwise or anticlockwise) scanning of the leaves in *T*. A circular order has the property that for any integer *k* (modulo *n*) all the branches on the path *P* (*x_k_*, *x_k+_*_1_) between *x_k_* and *x_k+_*_1_ correspond to the left branch (or right branch if anti-clockwise). Makarenkov and Leclerc (1997, 2000) have shown that the concept of circular ordering is equivalent to the so-called Yushmanov’s ordering (Yushmanov, 1984). A simple ordering algorithm can be used to order the leaves.

The algorithm starts by arbitrarily choosing two leaves *x*_1_ and *x_n_* and removing them from the set *X*. The resulting subset *W* is searched for the element *w* minimizing *d* (*x_n_*, *x_w_*) – *d* (*x*_1_, *x_w_*). This element takes the index (*n –* 1). The procedure is repeated after having removed the ordered leaf from the set *X* till all leaves are indexed. The (*n* – *k* – 1)th element is defined by the equation:

(3) $$\begin{array}{l} d(x_{n-k},\;x_{n-k-1})\;-\;d(x_{1},\;x_{n-k-1})\\ \;\;\;=\;{\text{min}}_{w}\;d(x_{n-k},\;x_{w})\;-\;d(x_{1},\;x_{w}) \end{array}$$

### 2b. Alternative method to the ordering problem

We present an alternative method to the ordering problem, a method that is appropriate to determine a circular order when the dissimilarity matrix does only approximatively correspond to an X-tree. Let us choose a leaf and index it with *n*. This leaf *n* is the reference leaf. For any two leaves (*i*, *j*), the distance $Y_{i,\;j}^{n}$ between the leaf *n* and the path *P* (*x_i_*, *x_j_*) is:

(4) $$Y_{i,j}^{n}\;=\;\frac{1}{2}(d(x_{i},\;x_{n})\;+\;d(x_{j},\;x_{n})\;-\;d(x_{i},\;x_{j}))$$

The distance matrix $Y_{i,\;j}^{n}$ has an underlying structure that can be used to determine a circular order of the leaves. The sets of leaves *X-x_n_* can be divided into two non intersecting subsets, the set of left leaves and the set of right leaves. Given a leaf *x*_1_, the set of leaves minimizing $Y_{1,\;j}^{n}$ is arbitrarily called the set *L* of left leaves. The set of right leaves corresponds to the set *X* – *L* – *x_n_*. A circular order is obtained by iteratively dividing the set of leaves *X* – *x_n_* into subsets of right and left leaves till only a single leaf is contained into each subset. At the first iteration, the set *X* is divided into two subsets *R*_1_ = {*x*_1_, …, *x_k_*_1_} and *L*_1_ = {*x_k_*_1+1_, …, *x_n–_*_1_} At the second iteration step, the set of right leaves (resp. left) is divided into right leaves *R*_1_*R*_2_ = {*x*_1_, …, *x_k_*_2_} and left leaves *R*_1_*L*_2_ = {*x_k_*_2+1_, …, *x_k_*_1_}. As the elements on the right leaves get the smaller indices, this procedure defines an order {*x*_1_, …, *x_n_*} for the set *X*. Once ordered with the above procedure, the distance matrix has $Y_{i,\;j}^{n}$ elements with values decreasing away from the diagonal.

#### Proposition 1

The leaves of an X-tree can be ordered so that the distance matrix $Y_{i,\;j}^{n}\;=\;1/2\;\cdot \;(d_{i,n}\;+\;d_{j,\;n}\;-\;d_{i,\;j})$ fulfils the inequalities $Y_{i,\;j}^{n}\;\geq \;Y_{i,\;k}^{n},\;Y_{k,\;j}^{n}\;\geq \;Y_{k,\;i}^{n}\;(i\;\leq \;j\leq \;k)$.

#### Proof

Let us order the elements in *X* – *x_n_* so that the elements in *R* are given lower indices than the elements in *L*. The resulting indexed distance matrix $Y_{i,\;j}^{n}$ can be divided into 4 submatrices. The two submatrices *RL* and *LR* with vertices belonging to different subsets (*x_i_* ∈ *R*, *x_j_* ∈ *L* or *x_i_* ∈ *L*, *x_j_* ∈ *R*) have elements all equal to a constant *K*_1_. The two other submatrices *RR* and *LL* (*x_i_*, *x_j_* ∈ *L* or *x_i_*, *x_j_* ∈ *R*) have all elements larger than the elements in *RL* and *LR*. If the above procedure is repeated iteratively on the right and left subtrees corresponding to the *R* and *L* subsets till one element is left in each subset then the inequalities $Y_{i,\;j}^{n}\;\geq \;Y_{i,\;k}^{n},\;Y_{k,\;j}^{n}\;\geq \;Y_{k,\;i}^{n}\;(i\;\leq \;j\leq \;k)$ hold.

The inequalities $Y_{i,\;j}^{n}\;\geq \;Y_{i,\;k}^{n},\;Y_{k,\;j}^{n}\;\geq \;Y_{k,\;i}^{n}\;(i\;\leq \;j\leq \;k)$ can be used to characterize the dissimilarity matrix and determine the degree to which the inequalities are satisfied by a given order. An order, with all above inequalities satisfied, is defined as a perfect circular order.

Let us define the contradiction level *C* of the order {*x*_1_, …, *x_n_*} as a weighted deviation to a perfect order:

(5) $$\begin{array}{l} C\;=\;\sum_{\begin{array}{l} k>\;j\geq i\\ i,j,k\neq n \end{array}}{\left(\text{max}\left(\left(Y_{i,\;k}^{n}\;-\;Y_{i,\;j}^{n}\right),\;0\right)\right)}^{\beta }\\ \;\;\;\;\;\;\;\;+\;\sum_{k\geq j>i}{\left(\text{max}\left(\left(Y_{i,\;k}^{n}\;-\;Y_{j,\;k}^{n}\right),\;0\right)\right)}^{\beta } \end{array}$$

which can be written as

(6) $$C\;=\;\sum_{i,\;j=1}^{n-1}C_{i,\;j}$$

The level of contradiction furnishes an optimization criterion to the ordering problem. If the dissimilarity matrix corresponds to an X-tree, then the minimum level of contradiction is zero (perfect circular order). When the dissimilarity matrix corresponds only approximately to an X-tree, the order minimizing *C* is per definition the order that is the closest to a perfect circular order.

## 3. Effect of Lateral Transfer and Crossover on Circular Order

### 3.1 Lateral transfer

**Figure 3.**
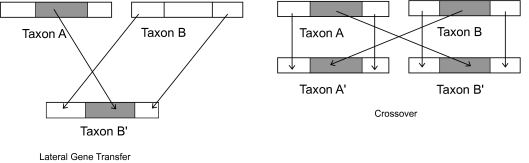
A lateral transfer from taxon A to taxon B corresponds to the replacement of some proportion *α* of taxon B by a segment of A. A crossover is characterized by an exchange of a portion *α* of genetic material between A and B.

In this section, we will discuss the possible influence of lateral transfer on circular order. Suppose that a proportion *α* of the genetic material of taxon A replaces some section of taxon B, as sketched in Figure 3. A lateral transfer can be modeled as a transformation of the distance matrix.

Most evolution models assume that the transition probability of each element in the sequence is independent. Evolution in one site does not affect the probability of changes in another site. For segments of equal lengths with a uniform distribution of the possible values and equal transition probabilities, the expected value of the distance between two leaves *δ̂_B, C_* is given by

(7) $${\hat{\delta }}_{B,C}\;=\;\alpha \cdot \delta_{A,C}\;+\;(1-\alpha )\cdot \delta_{B,C}$$

(8) $${\hat{\delta }}_{A,B}\;=\;(1-\alpha )\cdot \delta_{A,B}$$

with *δ_i,j_* the distance between the sequences indexed as *i* and *j* in the X-tree (i.e. without lateral transfer).

**Figure 4.**
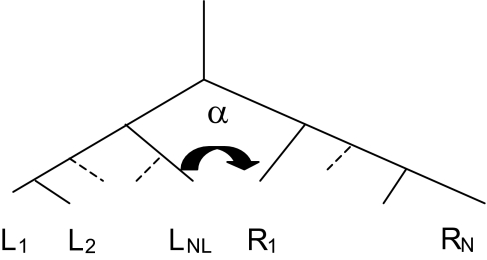
If a lateral transfer takes place between leaves that are consecutive then the expected distance matrix can be perfectly ordered.

Despite the fact that a lateral transfer suppresses the X-tree structure, some of the original tree topology can still be retrieved. In particular, we will show that if a lateral transfer takes place between two consecutive vertices, then the original order is preserved and the inequalities defining perfect order are still fulfilled by the transformed distance matrix (Fig. 4).

#### Proposition 2

Consider a lateral transfer from one leaf *A* to another leaf *B* of a valued X-tree. If the effect of the lateral transfer on the expected distance matrix can be modeled as *d̂_i,j_* = *d_i,j_* (*i, j* ≠ *B*), *d̂_B,C_* = α · *d_A,C_* + (1 – α) · *d_B,C_*, *d̂_A,B_* = (1 – α) · *d_A,B_* then there exists at least one circular order for which the expected distance matrix ${\hat{Y}}_{i,\;j}^{n}\;(n\;\neq \;A,\;B)$ satisfies the inequalities ${\hat{Y}}_{i,\;j}^{n}\;\geq \;{\hat{Y}}_{i,\;k}^{n},\;{\hat{Y}}_{k,\;j}^{n}\;\geq \;{\hat{Y}}_{k,\;i}^{n},\;(i\;\leq \;j\;\leq \;k)$, (*i* ≤ *j* ≤ *k*), characterizing a perfect order. In that order the two leaves *A* and *B* are consecutive (i.e. the two sequences are indexed with *i* and *i* + 1).

#### Proof

Before lateral transfer, the tree associated to the distance matrix $Y_{i,\;j}^{n}$ is a valued X-tree. For any X-tree, there is at least a circular order for which two arbitrary leaves are consecutive. Let us choose *A* and *B* to be consecutive in a circular order. After lateral transfer, the expected distances are related to the distances on the tree without lateral transfer by the equation: ${\hat{Y}}_{B,\;C}^{n}\;=\;\alpha \;\cdot \;Y_{A,\;C}^{n}\;+\;(1\;-\;\alpha )\;\cdot \;Y_{B,\;C}^{n}$ and ${\hat{Y}}_{A,\;B}^{n}\;=\;\alpha \;\cdot \;Y_{A,\;A}^{n}\;+\;(1\;-\;\alpha )\;\cdot \;Y_{A,\;B}^{n}$. As *Ŷ_B,C_* and *Ŷ_A,B_* are weighted averages on two consecutive sequences, the order is preserved and the distance matrix still satisfies the inequalities characterizing perfect order.

Corollary 1: If all lateral transfers can be ordered so that transfers are between consecutive leaves, then there exists at least one perfect order of the leaves.

Proposition 1 can be generalized to a lateral transfer between internal vertices using the following model. An *α – lateral transfer* from vertex *A* to vertex *B* is defined by the equations ${\hat{Y}}_{B,\;C}^{n}\;=\;\alpha \;\cdot \;Y_{A,\;C}^{n}\;+\;(1\;-\;\alpha )\;\cdot \;Y_{B,\;C}^{n}$ ((*A, C* ≠ *B, B_i_*) with *B_i_* the leaves descendant of the vertex *B*) together with the requirement that after an *α – lateral transfer*, (i) the matrix ${\hat{Y}}_{B_{i},\;B_{j}}^{n}$ can be ordered without contradiction, (ii) ${\hat{Y}}_{C,\;B_{i}}^{n}\;=\;{\hat{Y}}_{B_{i},\;C}^{n}\;\leq \;{\hat{Y}}_{B_{i},\;B_{j}}^{n}$ for *C* ≠ *B, B_i_*, *n* ≠ *B_i_*. (These conditions are fulfilled when the evolution of the section of *A* transferred to *B* can be described by an X-subtree)

Corollary 2: There exists at least one order of the leaves so that after an *α – lateral transfer* from vertex *A* to vertex *B* the distance matrix $Y_{C,D}^{n}$ is perfectly ordered.

The proof follows from proposition 2 together with the definition of an *α – lateral transfer*. Proposition 2 guarantees that the distance matrix restricted to the subsets of leaves including *B* but no descendant of *B* can be perfectly ordered. Replacing *B* by the ordered elements *B_i_* furnishes a perfect order.

**Figure 5.**
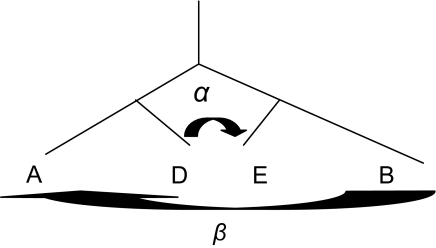
Perfect order cannot be guaranteed if there is no order for which taxa pairs involved in lateral transfer are consecutive.

Though corollary 1 gives a sufficient condition for the order (of the expected distances!) to be preserved, there are typical conditions involving more than two leaves for which no perfect order does exist. Figure 5 shows an example of lateral transfers that do generally not preserve order. This case is characterized by the fact that it is impossible to find an order of the sequences for which all sequences pairs involved in lateral transfers are consecutive leaves.

### 3.2 Crossover

A crossover is characterized by an exchange of a portion *α* of genetic material between *A* and *B* (Fig. 3). Under the same assumptions as in proposition 2, the expected distances can be modeled as ${\hat{Y}}_{B,\;C}^{n}\;=\;(1\;-\;\alpha )\;\cdot \;Y_{B,\;C}^{n}\;+\;\alpha \;\cdot \;Y_{A,\;C}^{n}$, ${\hat{Y}}_{A,\;C}^{n}\;=\;(1\;-\;\alpha )\;\cdot \;Y_{A,\;C}^{n}\;+\;\alpha \;\cdot \;Y_{B,\;C}^{n}$ and ${\hat{Y}}_{A,\;B}^{n}\;=\;Y_{A,\;B}^{n}$. If the two sequences *A* and *B* are consecutive and *α* ≤ 0.5 then there exists at least one order with *C* – *C_A,B_*= 0 (The proof is very similar to prop. 2). Contrarily to a lateral transfer this order is not always the order minimizing the contradiction.

### 3.3 Influence of lateral transfer and crossover on the order in a Jukes-Cantor model

The discussion in Section 3.1 on the robustness of perfect order against lateral transfer assumes that the true distance between taxa is correctly estimated. In real applications the evolutionary distance can only be estimated from data using a probabilistic model such as the Jukes-Cantor or Kimura 2-Parameter models. In the Jukes-Cantor model, evolution is described as a Markov process with a transition probability independent of the basis. The Jukes-Cantor model relates the distance between two sequences to the observed probability *p* for two sites to be different: *d* = *–*3/4 · ln(1 – 4/3 · *p*). Let us assume a model in which evolution proceeds according to the Jukes-Cantor model with the exception of a lateral transfer. In that case, the Jukes-Cantor model furnishes an estimation of the distance between the two sequences that often differs from the true distance. Nevertheless we can show that this estimation preserves the original order provided the two leaves are consecutive; proposition 2 is still valid when the distance is estimated by the Jukes-Cantor model. To show this, let us consider a lateral transfer from one leaf *A* to another leaf *B* of a valued X-tree. For an X-tree the effect of an *α*-lateral transfer on the expected distance matrix estimated by the Jukes-Cantor model is given by

(9) $${\hat{d}}_{B,C}\;=\;-3/4\cdot \;\text{ln}(1-4/3\cdot \;(\alpha \cdot \;p_{A,C}\;+\;(1-\alpha )\cdot \;p_{B,C}))$$

with *p_A,B_* the probability of two sites in *A* and *B* to be different. Writing

(10a) $$Y_{B,C}^{n}\;=\;-3/4\;\cdot \;(\text{ln}(1\;-\;Cn)\;+\;\text{ln}(1\;-\;Bn)\;-\;\text{ln}(1\;-\;BC))$$

(10b) $$Y_{A,C}^{n}\;=\;-3/4\;\cdot \;(\text{ln}(1\;-\;Cn)\;+\;\text{ln}(1\;-\;An)\;-\;\text{ln}(1\;-\;AC))$$

With *Xn =* 4/3 · *p_X_*_,_ *_n_*; *XY =* 4/3 · *p_X_*_,_*_Y_* (*X = A, B, C*). One gets

(10c) $$\begin{array}{l} {\hat{Y}}_{B,C}^{n}\;=\;-3/4\;\cdot \;(\text{ln}(1\;-\;Cn)\;+\;\text{ln}(1\;-\;\alpha \;\cdot \;An\;-(1\;-\;\alpha )\;\cdot \;Bn)\\ \;\;\;\;\;\;\;\;\;\;\;\;\;\;\;-\text{ln}(1\;-\;\alpha \cdot \;AC\;-\;(1\;-\;\alpha )\;\cdot BC)) \end{array}$$

Using the inequality *A/B* ≤ (α · *A* + (1 – α) · C)/(α · *B* + (1 – α) · *D*)) ≤ *C/D* which holds if A, B, C, D > 0 and *A/B* ≤ *C/D* one finds that

(10d) $$\begin{array}{l} (1\;-\;Bn)/(1\;-\;BC)\;\leq \;(1\;-\;\alpha \\ \;\;\;\;\;\times \;An\;-\;(1-\;\alpha )\;\cdot \;Bn)\;\leq \;(1\;-\;An)/(1\;-\;AC) \end{array}$$

and it follows that

$$\begin{array}{l} -\text{ln}((1\;-\;Bn)/(1\;-\;BC))\;\geq \;-\;\text{ln}((1\;-\;\alpha \;\cdot \;An\;-\;(1\;-\;\alpha )\\ \;\;\;\;\;\;\times \;Bn)/(1-\alpha \;\cdot \;AC\;-\;(1\;-\;\alpha )\;\cdot \;BC))\;\geq \\ \;\;\;\;\;\;-\text{ln}((1\;-\;An)/(1\;-\;AC)). \end{array}$$

From the last equation, one concludes that $Y_{B,\;C}^{n}\;\geq \;{\hat{Y}}_{BC}^{n}\;\geq \;Y_{A,\;C}^{n}\;=\;{\hat{Y}}_{A,\;C}^{n}$. If *A* and *B* are consecutive and $Y_{i,\;j}^{n}$ is ordered, then ${\hat{Y}}_{i,\;j}^{n}$ is therefore also ordered.

A similar discussion applies to crossover. The results in Section 3.2 also apply when the distances are estimated with the Jukes-Cantor model.

## 4. Algorithm for Contradiction Minimization

### 4.1 The Neighbor-Joining algorithm

The NJ algorithm contains two main steps that are repeated iteratively. For an X-tree, the NJ algorithm discovers, at each iteration step, two leaves that form a cherry in the tree. Once the two leaves are discovered, they are replaced by a new leaf. More precisely, in the first step, the two vertices (*i, j*) maximizing *r_i_* + *r_j_* – (*n* – 2) · *d*(*i, j*) (*r_i_* = ∑_*k*=1.*n*_ *d_i, k_*) are joined. In the second step the two leaves (*i, j*) are replaced by a new leaf *i* –*j*. The distance to another leaf *k* is obtained by averaging the distance between the leaves *i*,*j* and the leaf *k: d_i–j, k_* = 1/2 · (*d_i, k_* + *d_j,k_*). If the tree is not an X-tree, the NJ algorithm does not always preserve perfect order. Numerical simulations of a lateral transfer show that there are situations in which no permutation on the branches of the tree obtained with the NJ algorithm results into a perfect order, despite the fact that the distance matrix $Y_{i,\;j}^{n}$ can be perfectly ordered. The NJ algorithm is nevertheless very useful to initialize the search algorithm minimizing the contradiction level as will be seen below.

### 4.2 Algorithms for contradiction minimization

**Figure 6.**
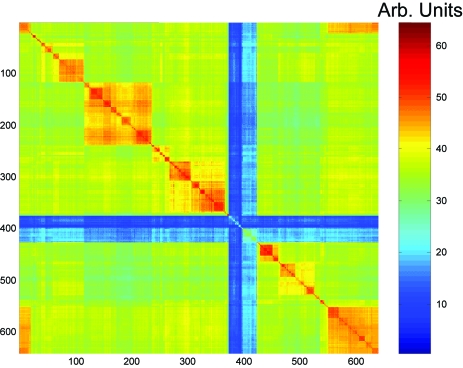
The search for the best order is initialized with an order compatible with the tree obtained by the NJ-algorithm. The color code is the same for all images. Large values of $Y_{i,\;j}^{n}$ are in red and small values in blue. The values are scaled to fit the scale.

In practical problems, the exact topology is unknown and some algorithms are necessary to find an order minimizing the contradiction. Solving the ordering problem is quite simple if the distance matrix $Y_{i,\;j}^{n}$ corresponds to a distance matrix *d* satisfying the 4-point relationship. In that case the ordering problem is a classical ordering problem for which several efficient algorithms are known. If the inequalities do not hold then the matrix may not be perfectly orderable. In that case, an order minimizing the contradiction level *C* is the best that can be achieved. Finding that order may require testing all possible permutations, a task requiring already for small problems a number of computations beyond the reach of computers. For that reason a search algorithm based on soft computing (Thuillard 2001) was designed. The first part of the search algorithm consists of initializing the order. The initial order is chosen to be compatible with the X-tree generated by the NJ algorithm (Fig. 6). This initial order is further refined by using a greedy algorithm. Restarting the NJ algorithm, the respective order of the two leaves joined by the NJ algorithm is chosen as the one minimizing the contradiction level of the X-tree. The last part of the search algorithm determines the “best” order by alternatively using the clustering and the optimization algorithm below. In that last phase, the topology of the NJ-tree is not taken as in the first optimization phases as a constraint on the search:

### Clustering Algorithm

1. Initialization: *v* (1) = 0.
2. For *i* = 1 … (*n* – 1) compute
  $$D(i)\;=\;\sum_{j=1}^{i+v(i)}Y_{i,\;j}^{n}/((v(i)\;+\;1)\;\cdot \;p)$$
  with
  $$p\;=\;\sum_{j=1}^{v(i)}\sum_{k=1}^{v(i)}Y_{i-j,\;i-k.}^{n}/v^{2}\;\text{if}\;v(i)\;>\;0$$
  and *p* = 0 else.
  If *D*(*i*) > Const then *v* (*i* + 1 ) = *v* (*i*) + 1 else (*v*(*i* + 1) = 1; *v*(*i*) = 0).
3. All successive sequences with *v*(*i* + 1) – *v*(*i*) = 1 are grouped into the same cluster.

### Optimization Algorithm

4. Randomly choose a cluster.
5. The cluster is inserted at the position reducing the most the contradiction value (computed on all sequences), defining so a new order of the sequences.

The optimization algorithm is quite efficient as only a small number of terms have to be calculated when the insertion point is shifted by one position.

Let us mention that the algorithm can be refined in many ways, using a more sophisticated clustering algorithm and a better search algorithm such as a multiresolution search algorithm (Thuillard 2001, 2004) or a simulated annealing. The algorithm can also be improved by optimizing the order within the clusters with the same hill climber. For the test data below, these refinements were found not necessary to obtain already a quite low level of contradiction.

## 5. Test on Real Data (SSU rRNA Archaea)

We will analyze below rRNA sequences for Archaea. In such sequences, lateral transfers or crossovers are believed to have had little influence on the evolution. In rRNA other deviations from the assumptions of the Jukes-Cantor model are the most likely cause of contradictions.

The algorithm was tested on the European ribosomal SSU rRNA database (Wuyts et al. 2004) using the entries for Archaea completed by the bacterial SSU rRNA for Aquificales and Thermotogales. The distance matrix was computed on the aligned data using the Jukes-Cantor model in the MBEToolbox (Cai et al. 2005). The order was optimized for low contradiction using the algorithm described in section 4. The reference taxon *n* was chosen among the bacterial rRNA.

The 2-dimensional representation of the SSU rRNA data permits to visualize proximity relationships between taxa. One can rapidly identify regions in which proximity relationships are essentially correct and regions with taxa not fitting well on an X-tree. Figure 6 shows the initial order for Archaea, order that is compatible with the X-tree generated by the NJ algorithm. The taxa are quite far from being perfectly ordered. If the taxa were perfectly ordered, the values of $Y_{i,\;j}^{n}$ should decrease away from the diagonal (In the color code the values should all drift towards blue).

**Figure 7.**
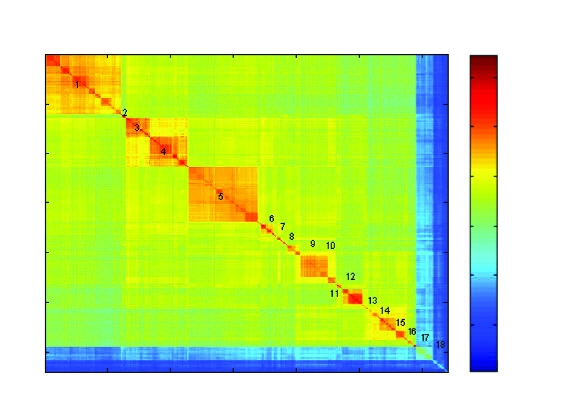
“Best” order obtained after the optimization. The numbers describe the different families and orders (see text for description).

After reordering with the algorithms described in section 4, the contradiction level is much lower. The main phylogenic orders are clearly identified (Fig. 7):

Reference sequence *n*: Thermotogales (str.-MV1075-AJ250439)

Euryarchaeota

1. Halobacteria (1–123)
2. Unidentified-Archaeon (124–129, AB01975*x,x* = 2, 3, 5, 6; AF05612, AF050620)
3. Methanococci Methanomicrobiales (130–167)
4. Methanococci Methanoarcinales (168–228)
5. Methanobacteria (229–341)
6. Unidentified-Archaeon (342–374)
7. Thermoplasmales (375–377)/unidentified Archaeon (378–385?)
8. Methanococci Methanococcales (386–405)
9. Thermococcus (406–450)
10. Archaeoglobi (453–461)
11. Methanopyrus Kandleri (462–463(?))

Crenarchaeota

12. “Crenarchaeotes” includes Cenarcheum Symbiotic (464–515)
13. Desulfurococcales (516–531)
14. Sulfolobales (532–557)
15. Thermoproteales (558–575)

Korarchaeota

16. Korarchaeota (576–589) together with unidentified-archaeon-AB01971x, (x =4,5,6).

Bacteria

17. Aquificales (590–614)
18. Thermotogales (615–640)

**Figure 8.**
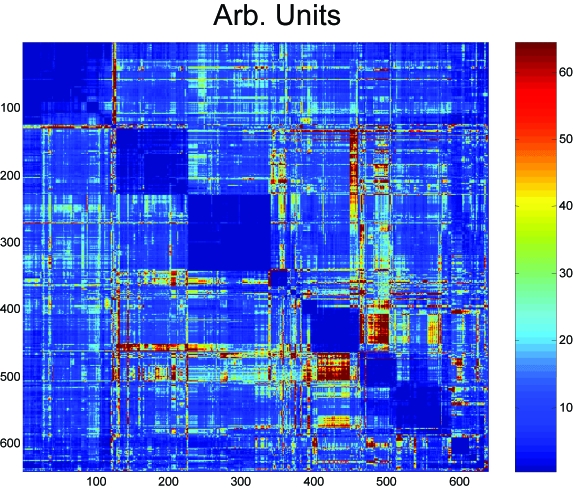
The level of contradiction *C*(*i,j*) associated to the “best” order of $Y_{i,\;j}^{n}$ in Fig. 7 is shown. The regions with high levels of contradictions are in red.

Contradictions are identified by plotting the values of *C* (*i, j*) in Figure 8. The level of contradiction is quite low. It can be measured with the following expression:

$$C_{norm}\;=\;C/(\sum_{j\geq i,k>j}{\left(Y_{i,k}^{n}\;-\;Y_{i,j}^{n}\right)}^{\beta }\;+\;\sum_{j>i,k\geq j}{\left(Y_{i,k}^{n}\;-\;Y_{j,k}^{n}\right)}^{\beta }).$$

After 200 iterations of the clustering and optimization algorithm described in section 4.2 (*β* = 2), C*_norm_* is typically below 0.007.

Figure 8 shows the level of contradiction of each pair (*i, j*) in the distance matrix. Many contradictions involve the clusters corresponding to Thermococcus (9) and Archaeoglobi (10) and Crenarchaeotes (12). The largest contradictions are between a mostly unidentified ensemble (12) of Crenarchaeotes including the marine sponge symbiotic Cenarcheum Symbiotic and Thermococcus (9).

**Figure 9.**
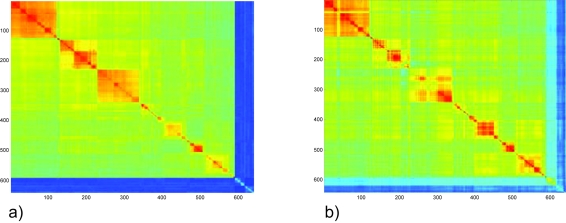
Representation of the distance matrix $Y_{i,\;j}^{n}$ with the order of Figure 7: (**a**) averaged over Aquificales and Thermotogales as reference element *n*; **b**) using Kimura-2 Parameter instead of Jukes-Cantor for computing $Y_{i,\;j}^{n}$.

Figure 9a shows the average over n of $Y_{i,\;j}^{n}$ obtained with the order of Figure 7, using 50 different reference taxa (25 Aquificales and 25 Thermotogales). No major difference is observed between both figures. Figure 9b shows the order of Figure 7 using Kimura-2 Parameter for computing the distance matrix $Y_{i,\;j}^{n}$. The level of contradiction is significantly higher. Even if some of the contradiction can be removed by further optimization, the level of contradiction stays higher than with the Jukes-Cantor model.

**Figure 10.**
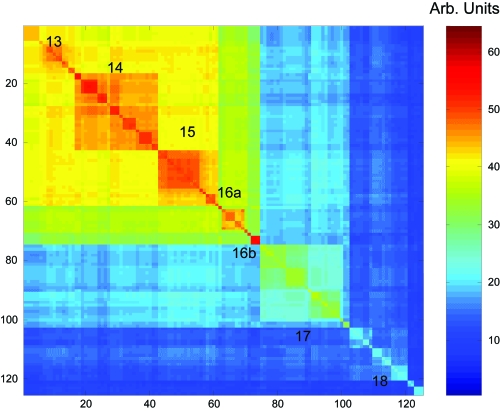
Zoom on the Crenarchaeota and Korarchaeota in Figure 7. The cluster 16a corresponds to Korarchaeota, while the cluster 16b corresponds to unidentified-Archaeon from some sulphur-emitting vents and deep hydrothermal sources. The sequences corresponding to 16b are the closest to the Aquificales and Thermotogales sequences.

Figure 10 focuses on the deepest branches corresponding to Korarchaeota and Crenarchaeota. Korarchaeota show a quite constant distance to Crenarchaeota, what is compatible with Korarchaeota being one of the deepest branch in Archaea. The deepest branch seems however occupied by taxa (Takai and Horikoshi, 1999). from some sulphur-emitting vents and deep hydrothermal sources (unidentified-archaeon-AB0197X, X = 14, … 17).

## Conclusion

A new method was developed to visualize phylogenetic relationships between genetic sequences. The visualization of the phylogenetic information is given by the 2-dimensional representation of the ordered distance matrix $Y_{i,\;j}^{n}=\;\frac{1}{2}(d(x_{i},\;x_{n})\;+\;d(x_{j},\;x_{n})-\;d(x_{i},\;x_{j}))$. The ordering algorithm uses a measure of the deviations from a perfect order to search for the order minimizing contradictions. An hybrid search strategy is followed: First an initial order is determined by a tree building method (Neighbor-Joining in our example). The order is then refined by using an optimization method (Hill climbing in our example, but it could also use a multiresolution search or genetic algorithms to give a few possibilities). The ordering algorithm has been tested on the rRNA sequences for Archaea. After optimization the sequences are grouped in different clusters. Regions with large deviations from a Jukes-Cantor model can be easily identified.

## Figures and Tables

**Figure 1.**
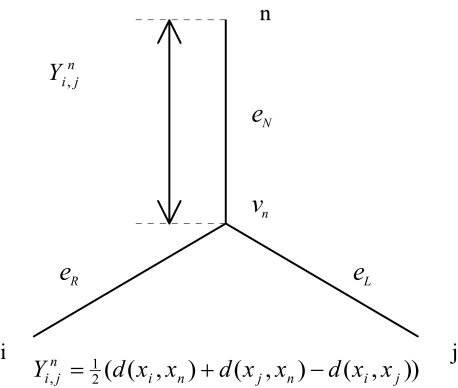
The distance matrix $Y_{i,\;j}^{n}$ corresponds to the distance between the leaf *n* and the path *P*(*i, j*).

**Figure 2.**
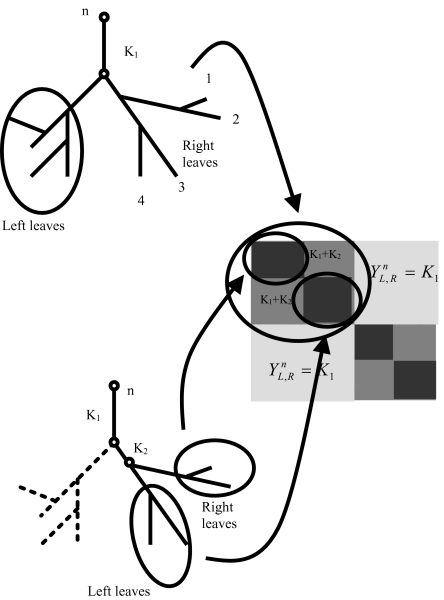
The leaves of an X-tree can be ordered so that distance matrix $Y_{i,\;j}^{n}$ fulfils the inequalities $Y_{i,\;j}^{n}\;\geq \;Y_{i,\;k}^{n}$ and $Y_{k,\;j}^{n}\;\geq \;Y_{k,\;i}^{n}\;(i\;\leq \;j\;\leq \;k)$.
